# Clinicopathological analysis of recurrence patterns and prognostic factors for survival after hepatectomy for colorectal liver metastasis

**DOI:** 10.1186/1471-2482-10-27

**Published:** 2010-09-27

**Authors:** Michihiro Hayashi, Yoshihiro Inoue, Koji Komeda, Tetsunosuke Shimizu, Mitsuhiro Asakuma, Fumitoshi Hirokawa, Yoshiharu Miyamoto, Junji Okuda, Atsushi Takeshita, Yuro Shibayama, Nobuhiko Tanigawa

**Affiliations:** 1 Department of General and Gastroenterological Surgery, Osaka Medical College Hospital 2-7 Daigaku-machi, Takatsuki City, Osaka 5698686, Japan; 2 Department of Pathology, Osaka Medical College Hospital 2-7 Daigaku-machi, Takatsuki City, Osaka 5698686, Japan

## Abstract

**Background:**

Hepatectomy is recommended as the most effective therapy for liver metastasis from colorectal cancer (CRCLM). It is crucial to elucidate the prognostic clinicopathological factors.

**Methods:**

Eighty-three patients undergoing initial hepatectomy for CRCLM were retrospectively analyzed with respect to characteristics of primary colorectal and metastatic hepatic tumors, operation details and prognosis.

**Results:**

The overall 5-year survival rate after initial hepatectomy for CRCLM was 57.5%, and the median survival time was 25 months. Univariate analysis clarified that the significant prognostic factors for poor survival were depth of primary colorectal cancer (≥ serosal invasion), hepatic resection margin (< 5 mm), presence of portal vein invasion of CRCLM, and the presence of intra- and extrahepatic recurrence. Multivariate analysis indicated the presence of intra- and extrahepatic recurrence as independent predictive factors for poor prognosis. Risk factors for intrahepatic recurrence were resection margin (< 5 mm) of CRCLM, while no risk factors for extrahepatic recurrence were noted. In the subgroup with synchronous CRCLM, the combination of surgery and adjuvant chemotherapy controlled intrahepatic recurrence and improved the prognosis significantly.

**Conclusions:**

Optimal surgical strategies in conjunction with effective chemotherapeutic regimens need to be established in patients with risk factors for recurrence and poor outcomes as listed above.

## Background

Despite recent improvements in the diagnosis and management of colorectal cancer (CRC), which have enabled early detection followed by early treatment, many advanced cases with hepatic or peritoneal metastasis are still encountered. For further improvement of the prognosis of CRC, it is particularly important to prolong the survival of these advanced cases with distant metastasis.

Hepatectomy for liver metastasis from colorectal cancer (CRCLM) is recommended as the most effective therapy [1-11]. However, to date, 50% to 75% of patients develop recurrence of the disease after curative resection of CRCLM. Moreover, the cure rate by initial hepatectomy is only 20% to 30% of cases [4-6,12]. Factors associated with recurrence and prognostic determinants after initial hepatectomy are still controversial. Recently, many studies reported that the duration of survival is markedly prolonged by effective chemotherapies, including the molecular target drugs, for unresectable or recurrent colorectal cancer [13,14]. Therefore, it is necessary to elucidate predictive factors relevant to recurrence and survival in order to determine optimal treatment strategies for each patient with CRCLM.

The aim of this study was to retrospectively investigate surgical outcomes in a consecutive series of patients undergoing hepatectomy for CRCLM at a single institution, in order to analyze recurrence patterns and clinicopathological prognostic factors for survival.

## Methods

### Patient selection

A retrospective study was conducted on 85 consecutive patients undergoing initial hepatectomy for CRCLM for curative intent at Osaka Medical College Hospital from 1995 to 2008. Clinical records and follow-up data were obtained for 83 patients (55 men, 28 women). The mean age at the initial hepatectomy was 66.5 ± 10.4 years (range, 29-87 years). The mean observation period was 62.8 months (range, 4-129 months). Perioperative mortality was not observed in patients in this study. All patients signed appropriate informed consent for surgical operation and to their inclusion in the study. The current study was approved by the ethics committee of Osaka Medical College Hospital. We also attest that this human study undertaken as part of the research from which this manuscript was derived is in compliance with the Helsinki Declaration.

### Characteristics of colorectal cancer

The primary tumor was in the colon in 60 cases (72.3%) and in the rectum in 23 cases (27.7%). Among patients with CRC, tumors were located in the right colon in 13 (15.7%), transverse colon in 9 (10.8%), and the left colon in 38 (45.8%). Sixty-three percent of the primary tumors had involved regional lymph nodes, and 46.8% and 51.9% had well- or moderately-differentiated histology, respectively. The histological depth of invasion in the colorectal wall is denoted as follows: se, serosa; ss, sub-serosa; a1, sub-adventitia; a2, adventitia. In 58 patients (71.6%), the depth of the primary tumors was within subserosal layer (≤ ss (a1)) of the colorectal wall.

### Hepatectomy

In this series, hepatectomy was indicated for CRCLM when the following 3 conditions were met: (1) the primary CRC was curatively resected; (2) metastasis located only in the liver; and (3) no limitation regarding the number or size of CRCLM as far as hepatic functional reserve was warranted after hepatectomy. All hepatectomies were performed by three experienced hepatobiliary surgeons (MH, FH, and NT) during the study period. All patients received potentially curative hepatectomy with removal of gross tumor with a negative macroscopic margin. With respect to hepatic hilar lymph nodes, lymph node dissection is not routinely performed, since node-positive cases in this region were strongly associated with extremely poor survival in our previous experience (data not shown).

Synchronous (as opposed to metachronous) CRCLM was defined as simultaneous presentation of liver metastasis at the time of CRC operation, and was detected in 28 patients (33.7%). They received either synchronous or metachronous hepatectomy, mainly based on the each patient's condition and need.

Generally, partial or non-anatomical hepatectomy was performed, whereas systemic or anatomical hepatectomy was preferred in cases when this procedure had advantage in terms of operation time, blood loss, safety, and invasiveness. Hepatic resection was performed following a standard technique as previously reported [15]. An ultrasonic dissector (SonoSurg system; Olympus Inc., Tokyo, Japan) was used for parenchymal transection, and small vessels were ligated or coagulated using soft-coagulation system or bipolar electrocautery. During the resection procedure, surgical margin was carefully confirmed using intraoperative ultrasonography in order to obtain a general surgical margin of 5-10 mm. Thus, in 63.6% of cases, hepatic surgical margin was wider than or equal to 5 mm.

### Chemotherapy

With respect to chemotherapy before hepatectomy and adjuvant chemotherapy after hepatectomy, the eligibility criteria in this series included histologically-proven adenocarcinoma of the colon or rectum. Patient criteria included an Eastern Cooperative Oncology Group (ECOG) performance status of 0-2. Additionally, patients had to have no serious or uncontrolled concurrent medical illness; no active infection; adequate hematologic parameters (WBC > 4.0 × 10^3^/L, platelet count > 100 × 10^9^/L), renal functions (serum creatinine ≤ 1.2 mg/dL or calculated creatinine clearance by Cockroft formula ≥ 50 mL/min), or hepatic functions (total bilirubin < 2.0 mg/dL and aspartate aminotransferase, alanine aminotransferase < 100 IU/L). Regimens consisted of 5-Fluorouracil (5-FU) alone, 5-FU/leucovorin (LV), 5-FU/cisplatin, tegafur plus uracil (UFT) alone, UFT/LV, oteracil (TS-1), FOLFOX (infusional 5-FU/LV + oxaliplatin), FOLFIRI (infusional 5-FU/LV + irinotecan) and IFL (5-FU/LV + irinotecan). Thus, 35 patients (42.2%) received chemotherapy before hepatectomy and 56 patients (71.8%) received adjuvant chemotherapy after hepatectomy.

### Patient follow-up

Patients were examined for CRCLM recurrence by ultrasonography and contrast enhanced computed tomography (CT) every 4-6 months and blood tests including tumor markers, such as carcinoembryonic antigen (CEA), every 2-3 months after discharge. When recurrence was suspected, magnetic resonance imaging was performed to ensure the appearance of new lesions in the remnant liver, while systemic recurrence was examined by fluorodeoxyglucose-positron emission tomography or Gallium scintigraphy. Chest and pelvic CT was also performed principally every 6 months for local and pulmonary metastasis or recurrence. Recurrence was diagnosed when at least two imaging studies confirmed the new lesions showing typical features of CRC/CRCLM, compared to the previous images. The recurrent CRCLM were treated by repeat hepatectomy when applicable (n = 15), otherwise by systemic chemotherapy, or their combination. During the study period, no major changes in clinical aspects were undertaken except for the use of more potent chemotherapeutic agents applied in the recent patients, with other surgical techniques and perioperative patient management being carefully conserved so as to minimize possible bias.

### Clinicopathological analysis

Patient demographics, laboratory tests including tumor markers, tumor characteristics, treatment, recurrence, and survival data were analyzed to determine prognostic factors in terms of 3- and 5-year survival rates after the initial hepatectomy for CRCLM. The surgically resected specimens were studied macro- and microscopically to determine the various tumor characteristics including size of the largest tumor, number of tumors, morphology and extent of the tumor, and surgical margin. For microscopic analysis, the resected specimens were fixed in 10% formaldehyde and sliced into 5-mm sections. After each section was sliced into 5 -μm tissue sections and stained with hematoxylin and eosin, two specialists of pathology (YS, AT) reviewed for histological confirmation of the pathological diagnosis. In this study, surgical margin status was defined as the distance of the lesion closest to the cut surface of the liver, and macroscopically classified into two categories: a surgical margin of 5 mm or wider (≥ 5 mm), and narrower than 5 mm (< 5 mm).

### Statistical analysis

Actuarial survival rate was calculated by the Kaplan-Meier method. Univariate analyses were performed using the log-rank test. Multivariate analyses were performed by Cox proportional hazards regression. Statistical comparisons were made by Fisher's exact probability test. A difference was regarded as statistically significant at *P *< 0.05.

## Results

### Primary colorectal tumor characteristics

Tumor characteristics of primary CRC were analyzed for prognostic values (Table 1). Included were: tumor location (colon or rectum), tumor differentiation (well, moderately, or poorly differentiated adenocarcinoma), number of lymph nodes metastasis, depth of tumor invasion in the colorectal wall (≤ ss (a1) or ≥ se (a2); ss, sub-serosa; se, serosa; a1, sub-adventitia; a2, adventitia), lymphatic invasion, venous invasion, and Duke's stage; the only significant difference in survival rate was observed between patients with primary CRC ≤ ss (a1) and those with ≥ se (a2) in the colorectal wall (*P *= 0.0133).

**Table 1:** Analysis of clinicopathological factors for prognosis

Factor	Number of patients	3-year survival rate (%)	5-year survival rate (%)	Univariate *P-value*	Multivariate *P-value*
**Sex**				*0.9217*	
Male	55	61.1	57.0		
Female	28	65.9	58.6		

**Location of CRC**					
Rt-hemicolon	13	38.1	38.1		
Transverse colon	9	75.0	56.3		
Lt-hemicolon	38	58.8	53.9		
Any colon	60	57.0	50.7	*0.0794*	
Rectum	23	81.3	81.3		

**Depth of CRC**				***0.0133***	*0.3183*
< ss (a1)	58	70.0	66.5		
≥ se (a2)	23	47.3	39.4		

**Tumor differentiation of CRC**				*0.4712*	
Well	37	70.4	65.0		
Moderate	41	53.3	48.0		
Poor	1	100.0	100.0		

**Lymphatic invasion of CRC**				*0.7469*	
Present	66	62.6	62.6		
Absent	14	73.5	45.9		

**Venous invasion of CRC**				*0.4259*	
Present	63	67.2	64.2		
Absent	18	55.6	41.7		

**Lymph node metastasis of CRC**				*0.6040*	
0	30	70.9	57.8		
1~3	39	64.0	64.0		
≥4	13	48.4	48.4		
Present	52	59.7	59.7	*0.6779*	
Absent	30	70.9	57.8		

**Duke's stage of CRC**				*0.4376*	
A	7	80.0	80.0		
B	11	60.6	22.7		
C	33	60.2	60.2		
D	30	65.0	65.0		

**Age at hepatectomy**				*0.5150*	
< 50 years	6	66.7	66.7		
≥50 years	77	64.5	57.0		

**CEA before hepatectomy**				*0.2441*	
< 5 ng/ml	27	53.9	49.4		
≥ 5 ng/ml	54	68.7	62.4		

**Time of liver metastasis**				*0.7865*	
Synchronous	28	61.2	61.2		
Metachronous	55	63.7	56.8		

**Timing of hepatectomy**				*0.4927*	
Synchronous	13	70.5	70.5		
Metachronous	70	61.5	55.2		

**Method of hepatectomy**				*0.4862*	
Anatomical	44	62.1	55.9		
Non-anatomical	39	63.2	59.0		

**Extent of hepatectomy**				*0.0875*	
< Lobectomy	62	67.1	63.8		
≥ Lobectomy	21	46.5	31.0		

**Number of CRCLM**				*0.3868*	
Solitary	47	69.3	61.0		
Multiple	36	51.8	51.8		

**Location of CRCLM**				*0.1882*	
Unilobar	63	68.9	62.6		
Bilobar	20	32.2	32.2		

**Size of CRCLM**				*0.9254*	
< 3 cm	42	71.3	62.9		
3 - 5 cm	26	51.8	51.8		
≥ 5 cm	14	50.4	50.4		

**Resection margin of CRCLM**				***0.0399***	*0.3917*
< 5 mm	28	47.8	41.8		
≥ 5 mm	49	70.4	64.5		

**Portal vein invasion of CRCLM**				***0.0074***	*0.2689*
Present	8	28.6	0		
Absent	74	66.2	63.4		

**Intrahepatic recurrence**				***0.0104***	***0.0051***
Present	32	44.6	32.5		
Absent	46	75.1	75.1		

**Extrahepatic recurrence**				***0.0217***	***0.0064***
Present	26	43.1	43.1		
Absent	54	69.4	63.0		

**Lung recurrence**				*0.1349*	
Present	18	39.9	-		
Absent	62	66.1	60.3		

**Chemotherapy before hepatectomy**				*0.0991*	
Present	35	51.1	43.8		
Absent	48	72.7	68.6		

**Chemotherapy after hepatectomy**				*0.3617*	
Present	56	63.6	60.3		
Absent	22	52.8	45.2		

CRC: colorectal cancer, CRCLM: liver metastasis from colorectal cancer, CEA: carcinoembryonic antigen.

### Time of liver metastasis and timing of hepatectomy

Among 83 patients receiving hepatectomy for CRCLM, 13 (15.7%) patients received synchronous liver and colorectal resection, and 70 (84.3%) patients received metachronous resection (Table 1). The 3- and 5-year survival rates were 70.5% and 70.5% in 13 patients with synchronous resection, 61.5% and 55.2% in 70 patients with metachronous resection. No differences in survival rates between these two groups were noted (*P *= 0.4927).

Synchronous CRCLM was detected at the time of CRC operation in 28 patients (33.7%). Hepatectomy was done synchronously in 13 and metachronously in 15 patients. The 5-year survival rate was 70.5% in 13 patients with synchronous resection, and 54.2% in 15 patients with metachronous resection. No differences in survival rates between these two groups were noted (*P *= 0.6547). Moreover, timing of hepatectomy was not associated with intra- and extrahepatic recurrence (*P *= 1.0000).

### Metastatic liver tumor characteristics

**Figure 1:**
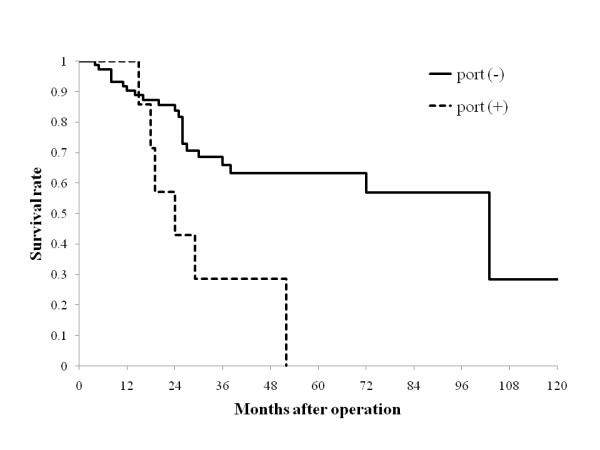
**Survival curves according to portal vein invasion in the resected liver specimen**. No portal vein invasive disease is denoted as "port (-)". Invasion confined to the portal vein is arbitrarily designated as "port (+)". Portal vein invasion was observed in 8 patients with the significant worse 3- and 5 year survival when compared with those without portal invasion (28.6% and 0% vs. 66.2% and 63.4%, respectively, *P *= 0.0074).

The mean and the median sizes of the largest metastatic lesions were 3.58 ± 1.93 cm and 3.0 cm, respectively. Of 83 patients, 47 patients (56.6%) underwent resection for solitary metastasis, 27 patients (32.5%) for 2 or 3 tumors, and 9 patients (10.8%) had 4 or more tumors resected (Table 1). In 63 patients (75.9%), the tumor location was unilobar (right in 36, left in 27), and 20 patients (24.1%) had bilobar disease resected. There were no significant differences in terms of the number, maximal size, and distribution of CRCLM (*P *= 0.3868, 0.9255, and 0.1882, respectively). The serum value of CEA immediately before hepatectomy was not associated with subsequent survival rate. Portal vein invasion was observed in 8 patients (9.8%) with the significantly worse 3- and 5-year survival when compared with those without portal invasion (28.6% and 0% vs. 66.2% and 63.4%, respectively, *P *= 0.0074; Figure 1). However, there was no significant correlation between portal vein invasion and intrahepatic recurrence rate (*P *= 0.7072).

### Operation-related parameters

With regard to the type of hepatectomy (anatomic, 44; non-anatomic, 39) and extent of resection (< lobectomy, 62; ≥ lobectomy, 21), no differences in survival rates between these groups were noted (*P *= 0.4862, 0.0875, respectively, Table 1).

**Figure 2:**
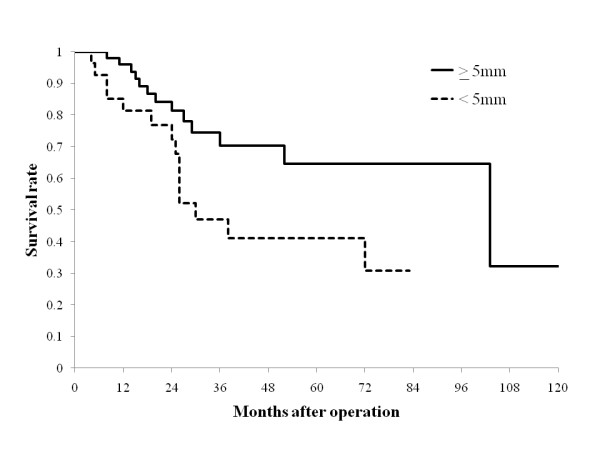
**Survival curves according to surgical margin in the resected liver specimen**. A surgical margin of 5 mm or wider is denoted as "≥ 5 mm". A surgical margin narrower than 5 mm is denoted as "< 5 mm". Patients with a surgical margin of 5 mm or wider had a better survival rate than those with a narrower resection margin (*P *= 0.0399).

Compared with the patients with a hepatic surgical margin ≥ 5 mm (n = 49, 63.6%), the 3- and 5-year survival rates for those with resection margin < 5 mm (n = 28, 36.4%) were significantly worse (≥ 5 mm vs. < 5 mm, 70.4% and 64.5% vs. 47.8% and 41.8%, respectively *P *= 0.0399; Figure 2). During hepatic parenchymal resection, tumors were exposed on the cut surface of the liver in 6 patients (7.3%); however, the survival of those patients was not significantly different from the other patients without tumor exposure.

### Intra- and extrahepatic recurrence after initial hepatectomy

Recurrence was detected in 52 (65.0%) of the patients who underwent hepatectomy (Table 1). The site of the first recurrence was the liver in 32 (41.0%) patients. Twenty-six (32.5%) patients had extrahepatic recurrence; the lung in 18, the peritoneal cavity or local recurrence in 9, the brain in 2, and the bone in 1. Patients with recurrent hepatic metastasis after initial hepatectomy (n = 37, 44.6%) had a significantly worse survival than those without hepatic recurrence (n = 46, 55.4%, *P *= 0.0104). Moreover, patients with recurrent extrahepatic metastasis after initial hepatectomy had a significantly worse survival than those without extrahepatic recurrence (*P *= 0.0217). Especially, those with lung metastasis after hepatectomy had extremely poor survival (3 years, 39.9%; 5 years, 0%).

Risk factors for intra- and extrahepatic recurrence were analyzed. On uni- and multivariate analysis, hepatic resection margin (< 5 mm) was significantly associated with intrahepatic recurrence after initial hepatectomy (*P *= 0.0133; Table 2). No significant risk factors for extrahepatic recurrence were identified (data not shown).

**Table 2:** Risk factors for intrahepatic recurrence after initial hepatectomy

Factor	Number of patients	Univariate *P-value*	Multivariate *P-value*
**Location of CRC**		*0.0733*	
Colon	27		
Rectum	5		

**Depth of CRC**		*0.2112*	
< ss (a1)	19		
> se (a2)	12		

**Tumor differentiation of CRC**		*0.4618*	
well	13		
moderate	16		
poor	1		

**Lymphatic invasion of CRC**		*1.0000*	
Present	25		
Absent	5		

**Venous invasion of CRC**		*0.7765*	
Present	23		
Absent	7		

**Lymph node metastasis of CRC**		*0.6320*	
Present	21		
Absent	10		

**Time of liver metastasis**		*0.8151*	
Synchronous	12		
Metachronous	20		

**Timing of hepatectomy**		*1.0000*	
Synchronous	6		
Metachronous	26		

**Number of CRCLM**		*0.3516*	
Single	16		
Multiple	16		

**Size of CRCLM**		*0.1218*	
< 5 cm	23		
≥ 5 cm	8		

**Portal vein invasion of CRCLM**		*0.7072*	
Present	4		
Absent	27		

**Surgical margin of CRCLM**		***0.0228***	***0.0133***
< 5 mm	15		
≥ 5 mm	13		

**Chemotherapy before hepatectomy**		*0.4837*	
Present	15		
Absent	17		

**Chemotherapy after hepatectomy**		*0.1976*	
Present	19		
Absent	12		

CRC: colorectal cancer, CRCLM: liver metastasis from colorectal cancer.

### Perioperative chemotherapy

Thirty-five patients (42.2%) received chemotherapy before initial hepatectomy, and 56 patients (71.8%) received chemotherapy after hepatectomy. Overall, the presence or absence of chemotherapy, regardless of chemotherapy before or after hepatectomy, or the combination, was not associated with intra- or extrahepatic recurrence or survival (Tables 1, 2). In subgroup analysis, in 55 patients with metachronous CRCLM, chemotherapy before and/or after hepatectomy was not associated with recurrence or the prognosis (Tables 3, 4). On the contrary, in 28 patients with synchronous CRCLM, adjuvant chemotherapy after hepatectomy was significantly associated with lower intrahepatic recurrence rate (*P *= 0.0087) and better prognosis (*P *= 0.0458) after initial hepatectomy, but not for extrahepatic recurrence (Tables 5, 6).

**Table 3:** Effect of peri-operative chemotherapy on survival for metachronous CRCLM

	Number of patients	3-year survival rate (%)	5-year survival rate (%)	Univariate *P-value*
**Chemotherapy before hepatectomy**				
Present	32	53.2	45.6	*0.1872*
Absent	23	80.0	72.8	

**Chemotherapy after hepatectomy**				
Present	36	57.9	53.1	*0.7919*
Absent	15	72.2	61.9	

**Table 4:** Effect of peri-operative chemotherapy on recurrence for metachronous CRCLM

		Intrahepatic recurrence		*P-value*	Extrahepatic recurrence		*P-value*
		
		Present	Absent		Present	Absent	
**Chemotherapy before hepatectomy**	Present	12	17	1.0000	13	18	*0.2394*
	Absent	8	13		5	16	

**Chemotherapy after hepatectomy**	Present	14	19	1.0000	13	22	*0.5335*
	Absent	6	9		4	11	

**Table 5:** Effect of peri-operative chemotherapy on survival for metachronous synchronous CRCLM

	Number of patients	3-year survival rate (%)	5-year survival rate (%)	Univariate *P-value*
**Chemotherapy before hepatectomy**				
Present	3	33.3	0	*0.2267*
Absent	25	66.0	66.0	

**Chemotherapy after hepatectomy**				
Present	20	76.5	76.5	***0.0458***
Absent	7	0	0	

**Table 6:** Effect of peri-operative chemotherapy on recurrence for synchronous CRCLM

		Intrahepatic recurrence		*P-value*	Extrahepatic recurrence		*P-value*
		
		Present	Absent		Present	Absent	
**Chemotherapy before hepatectomy**	Present	3	0	*0.0672*	0	3	*0.5360*
	Absent	9	16		8	17	

**Chemotherapy after hepatectomy**	Present	5	15	***0.0087***	8	12	*0.0681*
	Absent	6	1		0	7	

### Patient survival and prognostic factors

**Figure 3:**
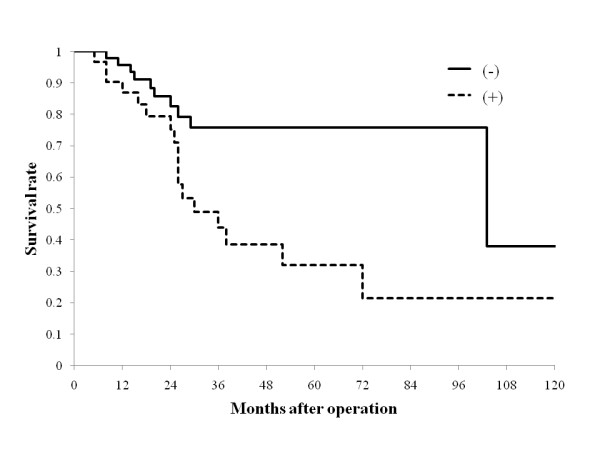
**Survival curves according to the presence of intrahepatic recurrence after the initial hepatectomy**. The presence of recurrence is denoted as "(+)" and absence "(-)". The presence of intrahepatic recurrence were associated with a significant difference in survival after initial hepatectomy (*P *= 0.0104).

The survival time ranged from 4 months to 129 months, mean survival time was 36.5 + 28 months, and median survival time was 25 months. The 5-year survival rate for the 83 patients after initial hepatectomy was 57.5%, which is comparable to reported survival rates of 20% to 58% [4,6-8,10-12,16,17]. Prognostic factors analyzed are shown in Table 1. On univariate analysis, tumor depth of CRC (≤ ss (a1) vs. ≥ se (a2)), portal vein invasion of CRCLM, macroscopic hepatic resection margin (< 5 mm vs. ≥ 5 mm), and the presence of intra- and extrahepatic recurrence were associated with a significant difference in survival rate after initial hepatectomy (*P *= 0.0133, 0.0074, 0.0399, 0.0104, and 0.0217, respectively, Table 1, Figure 3). Multivariate analysis revealed that independent prognostic factors for poor outcome were the presence of intra- and/or extrahepatic recurrence (*P *= 0.0051, 0.0064, respectively, Table 1).

**Figure 4:**
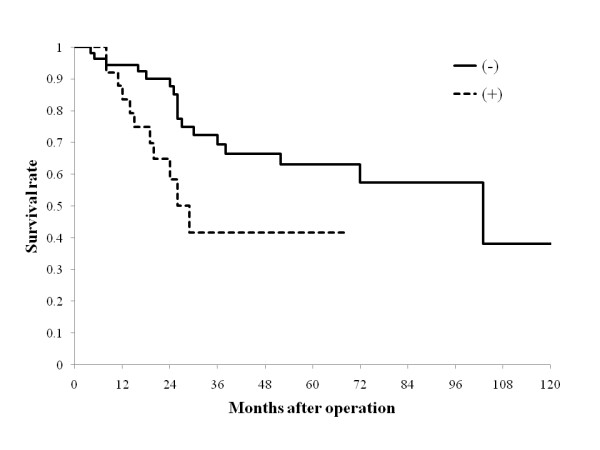
**Survival curves according to the presence of extrahepatic recurrence after the initial hepatectomy**. The presence of recurrence is denoted as "(+)" and absence "(-)". The presence of extrahepatic recurrence were associated with a significant difference in survival after initial hepatectomy (*P *= 0.0217).

## Discussion

While untreated CRCLM has a poor prognosis with median survival ranging from 6 to 12 months and is not expected to be over 5 years, long-term survival and potential for cure following surgical resection for CRCLM had been demonstrated in numerous uncontrolled studies [7,8,12]. These studies reported that the overall 5-year survival rates are in the range of 20% to 58%, and the median survival times are 24-46 months [4,6-8,10-12,16,17]. However, it has also been reported that 57% to 78% of those patients will develop a recurrence of the disease in the hepatectomy series, and intrahepatic recurrence occurs in approximately 50% of patients [4,6,9,18]. It has been considered that occult metastasis surfacing from the primary CRC and residual lesion scattered from liver metastasis are the two major pathways through which hepatic recurrence of metastatic lesion occurs after initial hepatectomy [19,20]. Therefore, treatment strategies including hepatic resection should be determined on the basis of these mechanisms for recurrence of metastasis. In Japan, surgical procedures for CRCLM are recommended if the following conditions are met: (1), the primary tumor was curatively resected; (2), metastasis is located only in the liver; (3), the patient is in a condition to be able to bear hepatectomy; and relative indications include: (4), extrahepatic metastasis can be controlled when present; and (5), reductive operation is a part of multimodal treatment [21]. However, the detailed consensus in terms of ideal timing, type, and extent of hepatectomy and optimal combination with perioperative chemotherapy are not currently provided with sufficient evidence. The aims of this study are to retrospectively evaluate the significant prognostic factors for survival and risk factors for recurrence in patients who underwent hepatectomy, and thereby to determine the optimal timing and method of hepatectomy with concurrent use of perioperative chemotherapy.

In the present study, prognostic factors on univariate analysis were the depth of the primary CRC, portal vein invasion and surgical margin at hepatectomy for CRCLM, and the presence of intra- and extrahepatic recurrence after the initial hepatectomy. Independent prognostic factors on multivariate analysis were the presence of intra- and extrahepatic recurrence. Of these factors, the portal vein invasion and surgical margin status at hepatectomy are important factors through which surgery can improve prognosis.

There have been several reports on the risk factors of intra- and extrahepatic recurrence [7,22-24]. In the present study, an independent risk factor for intrahepatic recurrence was the hepatic resection margin, while there were no risk factors regarding extrahepatic recurrence. Hematogenous dissemination of CRC was reported to be significantly associated with the size of liver metastatic nodules by the cascade theory [25]. In this theory, in accordance with the process of CRCLM metastasizing to the lung and subsequently to the other organs, the size of CRCLM is thought to increase. However, the present study did not include the size or the number of CRCLM as risk factors for intra- or extrahepatic recurrence, implicating the significance of surfacing micrometastasis from the primary CRC which could not be detected perioperatively.

The surgical margin of < 5 mm at hepatectomy was detected as a risk factor for subsequent intrahepatic metastasis in this study. The role of surgical margin status as a prognostic factor to predict posthepatectomy survival has been controversial [24,26-29], and in the largest single-center series, Are et al. reported that a margin width > 1 cm was an independent predictor for better survival and is optimal [28]. Nuzzo et al. also recently reported that a histological surgical margin of ≤ 5 mm was associated with lower overall and disease-free survival rates independent of other clinic-pathologic factors [29]. In contrast, Bodingbauer et al. demonstrated that the rate of recurrence at the surgical margin was low and a positive margin was not associated with an increased risk of recurrence either at the surgical margin or elsewhere, if hepatectomy was performed with ultrasonic dissector by experienced surgeons [24]. The present study is distinct from previous studies because macroscopic, instead of histological, surgical margin utilized as a parameter. A macroscopic surgical margin rather than microscopic margin was adopted, since 1) use of various dissection and coagulation devices, such as soft-coagulation system, ultrasonic dissector, and bipolar electrocautery during hepatic parenchymal transection [15], would potentially hinder an accurate assessment regarding pathological surgical margin status including R0 (no residual tumor) and R1 (microscopic residual tumor) status [24,29]; and 2) the macroscopic surgical margin was considered a better parameter of clinical usefulness for patient and treatment selection. Namely, during preoperative evaluation, whether a surgical margin of ≥ 5 mm was macroscopically feasible or not, it was utilized as a practical marker to determine which was indicated first as an initial therapy for each patient: hepatectomy or neoadjuvant chemotherapy. If the surgical margin was estimated to be < 5 mm, preoperative chemotherapy is the best option [30]. It does need to be noted that resection should not be precluded whatever the width of the surgical margin, since no other single treatment modality can surpass hepatectomy even with a 0- to 1-cm surgical margin [28]. In the present study, surgical margin status was determined as a prognostic factor on univariate, but not on multivariate analysis. These findings are in accordance with the report by Kokudo et al. that found the surgical margin was correlated with tumor recurrence but not with survival rate [26]. However, when an insufficient surgical margin is suspected during hepatectomy, or postoperative pathology of the resected specimen reveals inadequate surgical margin, adjuvant chemotherapy should be started to prevent intrahepatic recurrence, though a prospective study on this approach is necessary to make definite conclusions.

Portal vein invasion in the resected liver was observed in a small number of patients (9.8%), but also showed significant negative impact on survival, suggesting it could reflect the grade of aggressiveness and invasiveness of each tumor or the potential for intrahepatic micrometastases, as has been implicated by some authors [29,31] in the context of surgical margin status as a surrogate marker for malignant potential. Therefore, optimal surgery should include a strategy against portal vein invasion, such as anatomical hepatic resection including Glisson's sheath with a sufficient margin, and aggressive adjuvant chemotherapy for this subgroup of patients [32]. Further studies are needed to clarify its clinical significance.

Regarding the timing of hepatectomy for synchronous CRCLM, controversy remains on whether to perform simultaneous resection or to include an observation period for a couple of months. No differences between these two approaches were detected in the present study, suggesting that in synchronous situations, surgery should be planned at least before hepatic tumor(s) become unresectable and that patients need to be treated with adjuvant chemotherapy immediately, since this subgroup appeared to benefit from adjuvant chemotherapy.

A further point of concern is multiple bilobar CRCLM lesions which precipitate the most challenging situation for hepatic surgeons. Although some portion of this patient cohort would indeed benefit from aggressive chemotherapy and subsequent hepatectomy after successful down-sizing of those lesions, many of these patients are not candidates for surgical intervention. Appropriate application and adequate combination of modalities, such as radiofrequency ablation [33], percutaneous transhepatic portal embolization [34], and two-stage hepatectomy are expected to extend the surgical indication for these patients [35].

There is not yet a sufficient consensus as to whether the perioperative chemotherapy is significantly associated with disease-free survival or prognosis [36]. In the present report, a combination of surgical therapy and adjuvant chemotherapy after hepatectomy controlled intrahepatic recurrence and consequently improved the prognosis significantly, but only in the subgroup with synchronous CRCLM. Conversely, in the patient subgroup with synchronous CRCLM, adjuvant chemotherapy would be essential. As for metachronous disease, no benefits from chemotherapy were detected in this study. However, it should be noted that the chemotherapy in the present series mainly depended on UFT + LV, TS-1, FOLFOX and FOLFIRI, and the recently developed molecular targeting agents, such as bevacizumab and cetuximab, which have been reported to improve the prognosis of recurrent and unresectable CRC [14,37-39], were not included in this study. Since use of these newer chemotherapeutic agents before hepatectomy is expected to control recurrence by extermination of the micro cancer cell, a similar study is currently being conducted on those patients who received these newer chemotherapeutic agents.

## Conclusions

In CRCLM, intra- and extrahepatic recurrence were independent prognostic factors, and independent risk factors for intrahepatic recurrence included macroscopic resection margin during hepatectomy. Combination of surgery and adjuvant chemotherapy for synchronous CRCLM could control intrahepatic recurrence and significantly improve prognosis. Considering the outcomes of treatment for CRCLM are not yet satisfying, extermination of the micro cancer cell should be achieved by introduction of more potent chemotherapeutic agents in combination with optimal surgery. Further studies are needed to clarify this matter.

## Competing interests

The authors declare that they have no competing interests.

## Authors' contributions

MH conceived the study concept and design, was involved with patient care and drafted the manuscript and literature review. YI, KK, TS, MA, FH, YM, JO, AT, and YS were involved with formation of the study concept and design, patient care and drafting of the manuscript and literature review. NT carried out the operation on the patient and was the main contributor in the writing of the manuscript. All authors have read and approved the final version of the manuscript. Please see sample text in the instructions for authors.

## Pre-publication history

The pre-publication history for this paper can be accessed here:

http://www.biomedcentral.com/1471-2482/10/27/prepub
